# Metopimazine is primarily metabolized by a liver amidase in humans

**DOI:** 10.1002/prp2.903

**Published:** 2021-12-17

**Authors:** Robert W. Busby, Xiaokun Cai, Sihyung Yang, Luis Ramos, Lata Venkatarangan, Helen Shen, Stephen Wax, Abu J. M. Sadeque, Cyril De Colle

**Affiliations:** ^1^ Neurogastrx, Inc. Woburn Massachusetts USA; ^2^ QPS Newark Delaware USA

**Keywords:** aldehyde oxidase, amidase, D2, D3, dopamine receptor agonists, drug metabolism, gastroparesis, metopimazine, metopimazine acid

## Abstract

Metopimazine (MPZ) is a peripherally restricted, dopamine D2 receptor antagonist used for four decades to treat acute nausea and vomiting. MPZ is currently under clinical investigation for the treatment of gastroparesis (GP). MPZ undergoes high first‐pass metabolism that produces metopimazine acid (MPZA), the major circulating metabolite in humans. Despite a long history of use, the enzymes involved in the metabolism of MPZ have not been identified. Here we report a series of studies designed to identify potential MPZ metabolites in vitro, determine their clinical relevance in humans, and elucidate the enzymes responsible for their formation. The findings demonstrated that the formation of MPZA was primarily catalyzed by human liver microsomal amidase. Additionally, human liver cytosolic aldehyde oxidase (AO) catalyzes the formation of MPZA, in vitro, although to a much lesser extent. Neither cytochrome P450 enzymes nor flavin‐monooxygenases (FMO) were involved in the formation MPZA, although two minor oxidative pathways were catalyzed by CYP3A4 and CYP2D6 in vitro. Analysis of plasma samples from subjects dosed 60 mg of MPZ verified that these oxidative pathways are very minor and that CYP enzyme involvement was negligible compared to microsomal amidase/hydrolase in overall MPZ metabolism in humans. The metabolism by liver amidase, an enzyme family not well defined in small molecule drug metabolism, with minimal metabolism by CYPs, differentiates this drug from current D2 antagonists used or in development for the treatment of GP.

## Graphical abstract

Metopimazine acid is the predominant circulating metabolite, present at many fold the plasma concentrations of the parent, and yet the metabolic route to that metabolite has not been defined. Through a series of studies, we identified potential MPZ metabolites in vitro, determined their clinical relevance in humans, and elucidated the enzymes responsible for their formation.

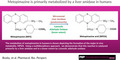

**Abbreviations**
ABT: 1‐aminobenzotriazole
AO: aldehyde oxidase
AUC: area under the concentration‐time curve
BNPP: bis‐p‐nitrophenyl phosphate
CYP: cytochrome P450
FAAH: fatty acid amide hydrolase
FMO: flavin monooxygenase
GP: gastroparesis
HKM: human kidney microsomes
HLC: human liver cytosol
HLM: human liver microsomes
KPi: potassium phosphate buffer
LC–MS: liquid chromatography‐mass spectrometry
MPZ: metopimazine
MPZA: metopimazine acid
MPZH: metopimazine hydroxylated metabolite
MPZS: metopimazine sulfoxide
NADPH: PAR, peak area ratio
UGT: uridine 5’‐diphospho‐glucuronosyltransferase

## 1 INTRODUCTION

Metopimazine (MPZ) is an approved drug in France under the brand name Vogalene^®^ (metopimazine, free base) that has been used for many years for the short‐term treatment of nausea and vomiting. A new salt formulation of MPZ is in clinical development in the US for the treatment of gastroparesis, a chronic disorder of the stomach characterized by delayed gastric emptying without evidence of mechanical obstruction.^1^, ^2^, ^3^ MPZ, a phenothiazine derivative, is a potent D 2/D3 selective, dopamine receptor antagonist that is peripherally restricted.^2^ The bioavailability of MPZ in humans is low. A 10 mg dose of MPZ was reported to have an absolute bioavailability under 20%.^4^ MPZ does not penetrate the blood–brain barrier and is subject to high first pass metabolism in humans.^4^, ^5^

While much is known about the pharmacology of MPZ, the metabolism of this drug is not fully elucidated. MPZ is deaminated to form metopimazine acid (MPZA), the major circulating metabolite, present at much higher plasma concentrations than the parent. At clinical doses of 20 and 50 mg of MPZ, MPZA accounted for 80% or more of the circulating drug‐related material in plasma following a single dose taken either preprandially or postprandially.^4^ This biotransformation is clearly the predominant metabolic pathway and yet the enzyme(s) responsible for this deamination reaction have not been determined. This metabolite retains some pharmacologic activity but is about 200‐fold less potent at the human D2 receptor compared to the parent.^2^ This underscores the importance of understanding this particular metopimazine metabolic transformation. Drugs or polymorphisms that could affect the enzymes involved in this biotransformation could significantly affect circulating metopimazine concentrations, which could affect both safety and efficacy. For example, phenothiazines at high enough doses, have been linked to drug‐induced orthostatic hypotension.^6^ Given the renewed interest in exploring the potential of MPZ for new clinical indications, identifying the enzyme(s) responsible for the MPZ to MPZA conversion is important to understanding any potential impact of metabolic variability on efficacy and safety risks and in assessing drug–drug interaction potential.

In this paper, we describe the multidisciplinary approach designed to determine the main human metabolites and elucidate the enzymes involved in MPZ metabolism. This entailed a thorough in vitro characterization of metopimazine metabolites derived from cellular and in vitro incubations as well as exploring the clinical relevance by looking for these metabolites in samples of healthy volunteers who were administered a 60 mg single dose of metopimazine. The limited role of cytochrome P450s in the metabolism of metopimazine has been more thoroughly defined. Most importantly, using a series of recombinant enzymes, inhibitor phenotyping and a variety of conditions, we identified the enzymes involved in the first pass metabolism of MPZ to form MPZA.

## 2 MATERIALS AND METHODS

The following experiments were performed in an exploratory manner.

### 2.1 Test article, metabolites and reagents

MPZ, MPZA, and metopimazine sulfate (MPZS) were supplied by Pacific Pharmaceutical Services. Bupropion, dextromethorphan, diclofenac, midazolam, paclitaxel, phenacetin, testosterone, itraconazole, montelukast, quinidine, sulfaphenazole, benzydamine, bis(4‐nitrophenyl) phosphate (BNPP), 7‐ethoxycoumarin (7‐EC), menadione, methimazole, phthalazine, and β‐nicotinamide adenine dinucleotide phosphate (NADPH, tetra sodium salt) were purchased from Sigma‐Aldrich. Chlorzoxazone and 1‐aminobenzotriazole (ABT) were purchased from Tokyo Chemical Industry. Coumarin was purchased from Acros Organics. (S)‐Mephenytoin, (+)‐benzylnirvanol, and tranylcypromine were purchased from Toronto Research Chemicals. Furafylline was purchased from Cayman Chemical. Pooled human liver microsomes (HLM, 150‐donor mixed gender) and human kidney microsomes (HKM, 5‐ donor mixed gender) were purchased from BioIVT. Pooled human liver S9 (50‐donor mixed gender) and human intestine S9 (10‐donor mixed gender) fractions and pooled human liver cytosol (HLC, 50‐donor mixed gender) were purchased from Xenotech. Supersomes™ CYP1A2, CYP2A6, CYP2B6, CYP2C8, CYP2C9, CYP2C19, CYP2D6, CYP2E1, CYP3A4, CYP3A5, FMO1, FMO3, and FMO5, were purchased from Corning Inc.. CTL P450 (Supersomes™ containing only P450 reductase and cytochrome b5) was also purchased from Corning, Inc., and used to normalize the protein content in the CYP reactions. Upon receipt, all test systems were stored at ≤−70°C until use.

### 2.2 Methods

#### 2.2.1 Preparation of reagents

Monobasic potassium phosphate (1 M) and dibasic potassium phosphate (1 M) stock solutions were diluted with water (Milli‐Q^®^, Millipore) to 100 mM. The 100 mM potassium phosphate buffer was prepared by adding the monobasic potassium phosphate solution (100 mM) to dibasic potassium phosphate solution (100 mM) until the pH value adjusted to 7.4, 8.4, or 9.4. The resulting 100 mM potassium phosphate buffer (KPi) was stored refrigerated (4°C). The NADPH solution (tetra sodium salt) was prepared fresh in KPi buffer prior to use and used in the reactions at a final concentration of 1 mM.

#### 2.2.2 Preparation of stock solutions

Stock solutions of MPZ at 20 mM (Lot No. AP237759063‐076‐01) were prepared in DMSO and stored at −20°C until use. Prior to incubations, 1 or 10 µM of MPZ (final concentration in the incubation mixture) was prepared. Primary stock solutions (Stock A) of positive control probe (7‐EC) were prepared in ethanol and stored at −20°C until use. The concentration of Stock A was 100 mM. On the day of the experiment, subsequent working solution of the positive control was prepared (from Stock A) at 100× the incubation concentration using appropriate buffer (Stock B).

Primary stock solutions (Stock A) of positive control probe (benzydamine and phthalazine) were prepared in DMSO and stored at −20°C until use. The concentration of Stock A was 100 mM (for benzydamine) and 1 mM (for phthalazine), respectively. On the day of the experiment, subsequent working solution of the positive control was prepared (from Stock A) in KPi buffer (Stock B).

#### 2.2.3 Clinical study

The clinical study was conducted at Biotrial Rennes, in accordance with the Declaration of Helsinki. The study (NG100‐101) was a double‐blind, placebo‐ controlled, ascending single‐ and multiple‐dose study of the safety, tolerability, and pharmacokinetics of metopimazine administered orally to healthy adult subjects. The study protocol, informed consent form, and appropriate related documents were approved by the institutional review board, and all subjects provided written informed consent prior to participation in the study.

In this study, sequential ascending single doses were administered to 4 cohorts of 8 participants (6 actives and 2 placebos per cohort, in cohorts 1, 2 and 3 following a randomization 6:2 and in cohort 4 with a 1:1 randomization for the two sentinel participants). In the morning, after at least 10 h of fasting overnight, eight healthy adult subjects (male or female aged 18 to 45), were orally administered metopimazine (or a matching placebo). Cohort 1–4 received a matching placebo or ascending doses of Vogalene (metopimazine) capsules at the doses of 15, 30, 45, or 60 mg, respectively. Blood samples were collected at the following time points: predose and 0.25, 0.5, 0.75, 1, 1.25, 1.5, 2, 3, 4, 6, 8, 12, 18, and 24 h postdose; plasma samples were obtained by centrifugation of the blood samples. A comprehensive safety and pharmacokinetic analysis was performed for each cohort for the SAD and MAD parts of the study and will be presented elsewhere. A fit‐for‐purpose discovery method was used to analyze 4 subjects from the highest single dosed cohort for the presence of metabolites.

Plasma concentrations of metopimazine, metopimazine acid and metopimazine sulfoxide were quantified using LC–MS/MS methods.

#### 2.2.4 Rat PK study

The pharmacokinetics of metopimazine (parent) and metopimazine acid (metabolite) were evaluated in male and female Sprague Dawley rats following a single oral dose of 45 mg/kg metopimazine (free base). Three animals per sex in each dose group were sampled at each timepoint after administration (0.25, 0.5, 1, 2, 4, 8, 12, and 24 h). The rats (rattus norvegicus) were obtained from Envigo RMS, Inc. and were 2 months old. Animals were weighed prior to each dose administration for the purpose of dose calculation. Whole venous blood samples of approximately 0.5 ml were collected from a peripheral vein of animals for determination of exposure to metopimazine and metopimazine acid. Three animals per sex were sampled at each timepoint. Samples were collected in tubes containing K2EDTA with protease inhibitor cocktail (Sigma, # P8340, at ratio 1:100) and placed on ice until processed for plasma by centrifugation under refrigeration for at least 10 min at 1912 x *g*. After centrifugation, supernatant was removed and stored frozen in a −70°C freezer until analyzed by LC–MS/MS.

#### 2.2.5 Dog PK study

The pharmacokinetics of metopimazine (parent) and metopimazine acid (metabolite) were evaluated in three male, beagle dogs following a single oral dose of 10 mg/kg metopimazine (free base). The dogs were obtained from Marshall BioResources. Animals were weighed prior to each dose administration for the purpose of dose calculation. Metopimazine was administered in size 13 gelatin capsules. Whole venous blood samples of approximately 1.0 ml were collected from a peripheral vein of all animals for determination of metopimazine and metopimazine acid. Samples were collected at the following timepoints: 0.25, 0.5, 1, 2, 4, 8, 12, and 24 h after test article administration. All animals were sampled at each timepoint. Samples were collected in tubes containing K2EDTA and placed on ice until processed for plasma by centrifugation under refrigeration for at least 10 min at 1912 x *g*. After centrifugation, the supernatant was removed and stored frozen in a −70°C freezer until analyzed by LC–MS/MS.

#### 2.2.6 Incubation of MPZ in human liver S9

An initial experiment was conducted with S9 fractions from liver and intestine to assess the role of liver and intestinal enzymes in the metabolism of MPZ. The reaction mixture contained 2.5 µM MPZ, pooled human liver or intestine S9 fraction (1 mg/ml S9 protein), 100 mM KPi buffer (pH 7.4). The reaction mixture was pre‐incubated for 3–5 min at 37°C in a humidified incubator filled with 5% CO_2_ followed by the addition of NADPH (1 mM) to initiate the reaction. The reaction was incubated for 0 and 60 min. The total percentage of organic solvent in the incubation mixture was ≤0.1%. The reactions were terminated by the addition of sample mixture of acetonitrile and ethanol (90:10, v/v). The positive control substrate, 7‐EC, was incubated in triplicate in an identical manner as MPZ for S9 fractions.

#### 2.2.7 Incubation with HLM

To conduct the metabolic incubation in liver microsomes in the linear range, protein concentration and the reaction time were optimized.

Optimization of protein concentration: For protein concentration optimization, MPZ at 1 µM was incubated with varying concentration of HLM proteins (0.1, 0.25, 0.5, and 1.0 mg/ml microsomal protein), 100 mM KPi buffer (pH 7.4). After pre‐incubation for 3–5 min at 37°C, the reaction was initiated by the addition of NADPH (1 mM) for 60 min in triplicates.

Optimization of Incubation Time: For incubation time optimization, MPZ at 1 µM was incubated with pooled HLM (0.5 mg/ml microsomal protein) and 100 mM KPi buffer (pH 7.4). After pre‐incubation for 3–5 min at 37°C, the reaction was initiated by the addition NADPH (1 mM) incubated for 0, 10, 20, 40, 60, and 90 min to determine the optimal incubation time (linear range). All metabolic reactions in this study were terminated by the addition of chilled organic solvent (acetonitrile:ethanol 90:10, v/v) containing internal standards, unless otherwise indicated.

#### 2.2.8 HLM Incubation with CYP inhibitors

To determine the role of microsomal CYP enzymes in the metabolism of MPZ, MPZ was incubated with pooled HLM at 1 and 10 µM in triplicate and the disappearance of MPZ and the formation of known metabolites (MPZA, MPZS, and MPZH) was assessed.

Initially, two sets of incubations were conducted with HLM (0.5 mg/ml microsomal protein), 1 and 10 μM MPZ, 100 mM KPi buffer (pH 7.4) where one set was in the presence and the other set was in the absence of 1 mM ABT, a non‐specific CYP inhibitor for 60 min. This allowed the assessment of total CYP contribution for MPZ metabolism. Then, CYP‐specific chemical inhibitors were used to assess the contribution of individual major CYP‐isoforms where HLM were incubated with 1 or 10 μM MPZ in the presence and in the absence of each CYP specific inhibitor at 37°C for 60 min. The following CYP‐isoform specific chemical inhibitors were used; furafylline (10 µM), tranylcypromine (10 µM), montelucast (0.5 µM), sulfaphenazole (5 µM), (+)‐benzylnirvinol (5 µM), quinidine (1 µM) and itraconazole (2.5 µM), for CYP1A2, CYP2B6, CYP2C8, CYP2C9, CYP2C19, CYP2D6, and CYP3A4/5. Parallel incubation with seven CYP specific probe substrates (phenacetin for CYP1A2, bupropion for CYP2B6, paclitaxel for CYP2C8, diclofenac for CYP2C9, S‐mephenytoin for CYP2C19, dextromethorphan for CYP2D6 and midazolam for CYP3A4/5) were included in as positive controls. For negative control reaction, MPZ was incubated at 37°C in duplicate in a reaction mixture devoid of NADPH. All other procedures were the same as described previously.

#### 2.2.9 Incubation with recombinant CYP‐isoforms

Recombinant CYP isoforms (Supersomes™) were used to determine the contribution of CYP isoforms in the metabolism of MPZ. MPZ at 1.0 µM was incubated in triplicate with individual CYP Supersomes™ (CYP 1A2, CYP2A6, CYP2B6, CYP2C8, CYP2C9, CYP2C19, CYP2D6, CYP2E1, CYP3A4, and CYP3A5). The final P450 content in the incubation mixtures was 50 pmol/ml. Protein concentrations for all CYP isoforms were normalized to the recombinant CYP containing the highest protein concentration. The reaction mixture containing MPZ or CYP substrates, KPi buffer (pH 7.4), and CYP‐ enzyme was pre‐incubated at 37°C for 3–5 min. All other procedures were the same as described previously. The marker substrates (coumarin for CYP2A6, chlorzoxazone for CYP2E1, midazolam for CYP3A4 and CYP3A5 and probe substrates for other CYP isoforms same as indicated in Section 2.2.8) were incubated at 1.0 µM in parallel. The negative control for incubation reactions incubated in the absence of NADPH under identical conditions.

#### 2.2.10 Incubation with HLM and HKM for FMO

MPZ, at 1.0 and 10.0 µM, was incubated with HLM or HKM to determine the contribution of microsomal FMOs on the metabolism of MPZ.

A reaction mixture containing KPi buffer (pH 8.4), HLM or HKM (0.5 mg/ml protein) and MPZ (1.0 and 10.0 µM) was pre‐incubated for 3–5 min at 37°C. The reaction was initiated by the addition of NADPH into the reaction mixture. The reaction was conducted for 0 and 60 min. A positive control (benzydamine, 100 µM) was incubated in duplicate for 0 and 20 min. Parallel incubation was conducted by adding 1 mM methimazole, a non‐specific FMO inhibitor, under identical conditions. Heat‐inactivated microsomes (50°C for at least 5 min) was incubated under identical conditions negative control. All other procedures were the same as described previously.

#### 2.2.11 Incubation with recombinant FMO

MPZ at 1.0 µM was incubated in triplicate with individual recombinant FMO (Supersomes™ for FMO 1, FMO3, and FMO5). Protein concentrations for all rFMO isoforms were 0.05 mg/ml. A positive control (benzydamine, 100 µM) was incubated for 20 min. The reaction mixture containing MPZ or FMO substrate, KPi buffer (pH 8.4), and FMO Supersomes™ was pre‐incubated at 37°C for 3–5 min. All other procedures were the same as described previously. The negative control reactions were incubated absence of NADPH.

#### 2.2.12 Incubations with HLM for microsomal hydrolase/amidase activity

Prior to the reaction for amidase activity, the reaction mixture containing KPi buffer (pH 7.4, 8.4, or 9.4) and HLM was pre‐incubated at 50°C for 5 min to inactivate CYP and FMO activities in HLM. The mixture was placed on ice to cool down. An aliquot was taken out which was considered as *t* = 0 min incubation. Then 10 µM MPZ was added into the remaining mixture and incubated at 37°C on an orbital shaker for 60 min. A parallel set of incubations was conducted with 10 µM MPZ in the presence at 2 and 16 µM, of BNPP (a known inhibitor of amidase). All reactions were in triplicate. The total percentage of organic solvent in the incubation mixture was ≤0.1%. The reactions were terminated by the addition of chilled acetonitrile: methanol, 90:10, (v/v). The samples were vortex‐mixed and stored at ≤−70°C and were subjected analyzed by LC–MS/MS.

#### 2.2.13 Incubations with HLC fractions for AO activity

The metabolism of MPZ was evaluated in the human liver cytosolic incubation. A metabolic incubation was performed with HLC (1 mg/ml protein) and 10 µM MPZ at 37°C for on an orbital shaker for 60 min. A parallel set of incubations were also conducted 10 µM MPZ in the presence of 100 µM menadione, a known inhibitor of human aldehyde oxidase. Another set of incubation was also conducted 1 µM phthalazine, a known substrate for aldehyde oxidase as positive control, under identical conditions.

#### 2.2.14 Preparation of human plasma samples

Venous blood samples (6 ml) for the determination of plasma concentrations of MPZ and metabolites (MPZA and MPZS) were drawn by direct venipuncture or via an intravenous catheter into K2EDTA Vacutainer^®^ tubes. It was important to avoid hemolysis during blood collection. After collection, the blood samples were centrifuged within 60 min, at 3000 rotations per minute for 10 min at 5°C. The resulting plasma was separated into 2 equal aliquots of 1000 µl (aliquots A & B) and transferred to polypropylene tubes, which were labeled, frozen within 120 min of blood collection and stored at –70°C until required for bioanalysis. Immediately prior to bioanalysis, samples were thawed and a 200 µl aliquots were removed and analytes extracted using acetonitrile protein precipitation. The extracted samples were analyzed twice, once for quantification of MPZ and MPZA and again for MPZS quantification as described below.

#### 2.2.15 LC/MS/MS method for metopimazine and metopimazine acid in rat, dog, and human plasma samples

An MRM LC–MS/MS method was developed for MPZ and metopimazine acid (MPZA) in rat, dog, and human plasma. The method was run in reverse phase in positive ion mode. Using an LC–MS/MS system that consisted of a Shimadzu Nexera X2 LC‐30 UPLC system (Shimadzu Corporation), and an API 4000 triple quadrupole mass spectrometer (SCIEX) running Analyst software 1.4. LC separation conditions were achieved for MPZ and MPZA using a Supelco Discovery HS F5 50 × 4.0 mm 3 µm column (Sigma‐Aldrich). D6‐isotopically labeled internal standards were used for each analyte. The following m/z transitions were monitored: metopimazine 446.3 → 141.1 and metopimazine acid 447.2 → 142.1. A calibration curve was generated for the quantification of MPZ and MPZA.

#### 2.2.16 LC/MS/MS method for metopimazine sulfoxide in human plasma

An MRM LC–MS/MS method was developed to look for metopimazine sulfoxide (MPZS) in human plasma. The method was run in reverse phase in positive ion mode. The system was identical to what was described above except that a slightly longer column was used, a Supelco Discovery HS F5 100 × 4.0 mm (3 µm) column (Sigma‐Aldrich). A D6‐isotopically labeled internal standards was used for MPS and the m/z transition monitored was 462.3 → 98.1. A calibration curve was generated for the quantification of MPZS in human plasma.

#### 2.2.17 LC/MS/MS method for in vitro samples

LC separation conditions were developed for MPZ and its metabolites (MPZA and MPZS), 7‐hydroxycoumarin, each metabolite of CYP probe substrate, benzydamine N‐oxide, and phthalazine. The LC–MS/MS system consisted of a Shimadzu Nexera X2 LC‐30 UPLC system (Shimadzu Corporation), and an API 4000 triple quadrupole mass spectrometer (SCIEX) was used for analysis. All analytes were analyzed using electrospray ionization (ESI) in positive ion mode except for 7‐EC, which used negative ion mode. The LC–MS/MS system was controlled by Analyst software (version 1.6.1, SCIEX).

For each analytical method, a calibration curve was generated for the quantification of MPZ and its two metabolites (MPZA and MPZS). Due to lack of the reference standard, the hydroxylated metopimazine (MPZH) was determined qualitatively based on peak area ratio (PAR) of presumed hydroxylated analyte to the IS. PAR was also used for all positive control compounds.

#### 2.2.18 Data analysis

The degree of disappearance of each analyte was evaluated by comparing the PAR of analyte to IS or concentration in different sampling time points to the PAR of time zero, (*t* = 0, 100%). The percent of parent remaining values were calculated according to the following equation:

$$\begin{array}{c} \begin{array}{c} \%\;\text{parent}\;\text{remaining}=(\text{mean}\;\text{concentration}\;\text{or}\;\text{PAR}\;\text{at}\;\text{each}\;\text{sampling}\;\text{time}\;\\ \text{point}/\text{mean}\;\text{concentration}\;\text{or}\;\text{PAR}\;\text{of}\;\text{time}\;\text{zero})\;\times 100 \end{array} \end{array}$$

All statistics (e.g., mean, SD) presented in the data tables are based on “precision as displayed” numbers (MS Excel, version 2010). GraphPad Prism (version 8) was used for figure generation.

Noncompartmental Pharmacokinetics analysis was performed in Phoenix 64 Winnonlin version 8.1.

#### 2.2.19 Nomenclature of targets and ligands

Key protein targets and ligands in this article are hyperlinked to corresponding entries in http://www.guidetopharmacology.org, the common portal for data from the IUPHAR/BPS Guide to PHARMACOLOGY,^7^ and are permanently archived in the Concise Guide to PHARMACOLOGY 2019/20.^8^

## 3 RESULTS

### 3.1 Metabolism in liver and intestinal S9 fraction, and microsomes

Upon incubation of MPZ with HLM, three primary metabolites were found on full scan LC–MS analysis. Based on peak area, the largest signal was found at m/z 447.14, which corresponded to MPZA, the major in vivo metabolite in humans. Two other isobaric peaks were found at m/z 462.15, consistent with MPZH and MPZS metabolites (Table 1). In human liver S9 fractions, approximately 30% of MPZ disappeared and all three metabolites (MPZA, MPZH and MPZS) were formed after a 60‐min incubation. In contrast, human intestine S9 fractions showed virtually no metabolism of MPZ, indicating intestinal metabolism is unlikely. HLM incubations were optimized for protein concentration and time based on the disappearance of MPZ and determined based on PAR. MPZ (1 μM) was incubated with HLM at various concentrations indicated in Section 2.2.7 for 60 min. An HLM protein concentration of 0.5 mg/ml were chosen for time optimization incubation. MPZ was incubated in HLM at 0.5 mg/ml protein concentration over 90 min. MPZ disappearance was essentially linear over the incubation period and there were 37.2% remaining at 60 min incubation time, which was considered optimal for future incubations. Authentic standards of MPZ, MPZA, and MPZS were available for quantification, while MPZH could only be semi‐quantified using peak area ratio due to lack of a synthetic standard.

**TABLE 1:** In vitro metabolites found following metopimazine incubations in human liver microsomes

Compound	Likely metabolite	Observed precursor ion (m/z)	Observed retention time (min)	Peak area at time 0	Peak area at time 90 min
MPZ—parent	—	446.15	4.01	2373	715
	MPZH	462.15	2.8	0	100
	MPZS	462.15	3.15	0	59
	MPZA	447.14	4.26	0	208

### 3.2 Clinical pharmacokinetics

**FIGURE 1:**
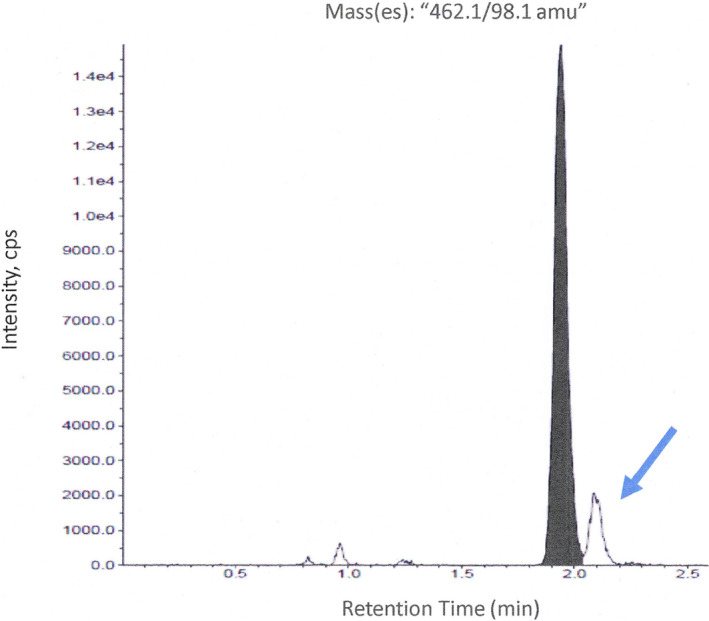
A representative total ion chromatogram monitoring the 462.3 → 98.1 m/z transition to quantify MPZS. An unidentified isobaric peak was observed in some samples (marked with arrow)

In vitro metabolism experiments had identified three potential metabolites, MPZA, MPZS, and MPZH. Bioanalytical methods were developed to quantify MPZ, MPZA, and MPZS in human plasma. The MPZH metabolite is isobaric with MPZS and it is separated enough from MPZS that it is visible in the same method in in vitro experiments (qualitative only). To look for the presence of these metabolites in vivo, plasma was sampled over 24 h from 4 subjects from the highest SAD cohort. Following a single oral dose of 60 mg of metopimazine, the mean clinical exposure parameters are provided in Table 2. As expected, the major circulating metabolite was MPZA, with Tmax ranging from 1.25–3 h and this analyte was present at all time points. MPZS was also found in all 4 subjects, though at much lower concentrations. It appeared at 30–45 min and was not quantifiable by 12 h. A representative chromatogram is shown in Figure 1. There was a very small isobaric peak that eluted just after 2 min (see arrow in Figure 1) in some of the samples. While it is speculative, this could potentially be the MPZH metabolite, albeit present at only trace amounts and unlikely to be quantifiable even if a standard could be made. A rough estimate of percent circulating drug‐related material of the verified in vivo metabolites based on AUClast is shown in Table 3. Based on this, MPZA accounts for approximately 90% of circulating material whereas the parent is less than 10%. Also, MPZS is a minor metabolite present at less than 1% of total circulating drug‐ related material.

**TABLE 2:** Mean pharmacokinetic exposure parameters of metopimazine and its human metabolites following a single 60 mg clinical dose

Subject	MPZ dose (mg)	MPZ			MPZA			MPZS		
		*C*_max_ (ng/ml)	*T*_max_ (h)	AUClast (ng‐h/ml)	*C*_max_ (ng/ml)	*T*_max_ (h)	AUClast (ng‐h/ml)	*C*_max_ (ng/ml)	*T*_max_ (h)	AUClast (ng‐h/ml)
1401	60	38.9	3	111.9	241	3	1592.6	1.6	4	7.6
1404	60	203	0.75	278.2	601	1.25	2506.1	10.2	1.25	25.5
1405	60	75.6	0.75	129.4	357	1.25	1941.9	3.1	1	10.0
1408	60	58.2	1	167.5	390	1.5	2265.8	4.6	12.5	20.1
Mean	60	93.9	1.4	171.8	397.3	1.8	2076.6	4.9	4.7	15.8
SD	—	64.3	0.9	64.7	130.0	0.7	343.8	3.3	4.7	7.3

**TABLE 3:** Percent drug‐related material following a single 60 mg clinical dose based on AUClast

Subject	MPZ dose (mg)	% Drug‐related material		
		MPZ	MPZA	MPZS
1401	60	6.5	93.0	0.4
1404	60	9.9	89.2	0.9
1405	60	6.2	93.3	0.5
1408	60	6.8	92.4	0.8
Mean	60	7.4	92.0	0.7
SD	—	1.7	1.9	0.2

### 3.3 Nonclinical pharmacokinetics

Given that MPZA was the predominant circulating metabolite in humans, bioanalytical methods were developed to quantify both parent and the major human metabolite. To determine the pharmacokinetic properties of metopimazine and its acid metabolite in rats and dogs, single doses of metopimazine (free base) were administered orally to each species and plasma samples were taken over time and analyzed. A noncompartmental analysis of the bioanalytical data was performed and the pharmacokinetic parameters determined. Table 4 shows the mean PK exposure parameters in rats and dogs.

**TABLE 4:** Mean (standard deviation) nonclinical pharmacokinetic exposure parameters after a single dose of metopimazine

Species (sex)	Metopimazine dose (mg/kg)	Analyte	*C*_max_ (ng/ml)	*T*_max_ (h)	AUC_inf_ (ng‐h/ml)
Rat (male)	45	MPZ	1076 (92)	4 (0)	8174 (2106)
		MPZA	89 (13)	4 (0)	755 (292)
Rat (female)	45	MPZ	1733 (306)	4 (0)	13914 (2060)
		MPZA	110 (20)	3 (1.7)	884 (156)
Dog (male)	10	MPZ	416 (241)	0.83 (0.29)	2600 (486)
		MPZA	103 (54.2)	2.0 (0.0)	728 (290)

Interestingly, the MPZA metabolite to parent ratio is far lower in dogs and particularly lower in rats than in humans, indicating MPZA is disproportionately produced in humans.

### 3.4 Cytochrome P450 involvement

**FIGURE 2:**
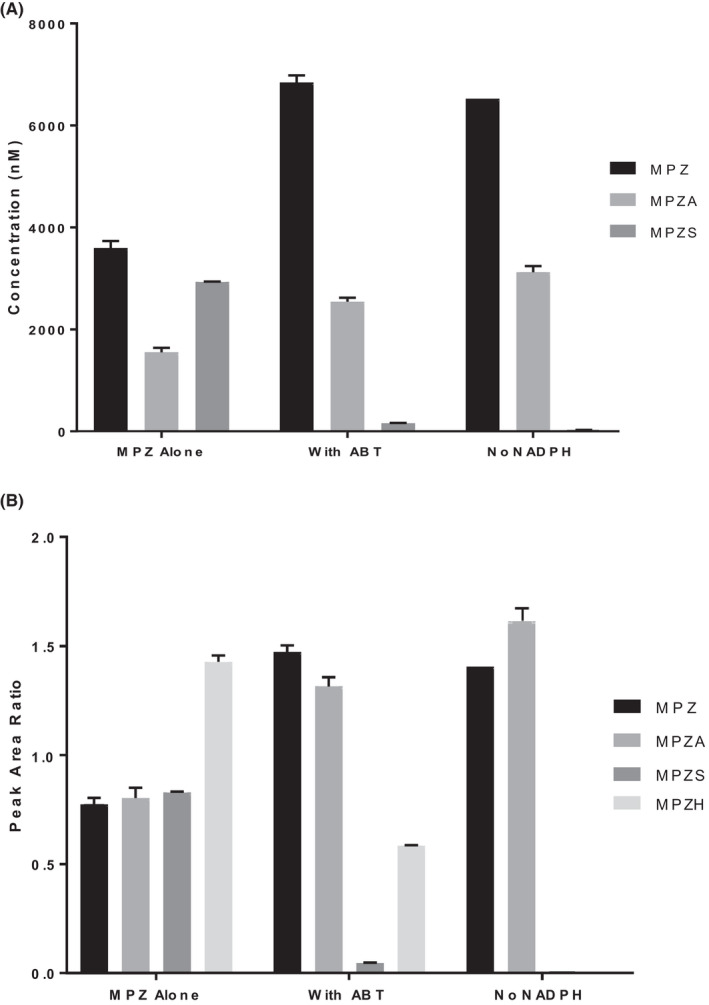
(A) (Upper) Mean (±SD) concentrations of MPZ, MPZA, and MPZS following incubations of 10 µM metopimazine with pooled human liver microsomes with NADPH and no inhibitor, with NADPH in the presence of 1‐ABT, a pan‐cytochrome P450 inhibitor, and without NADPH or inhibitor in the incubation. MPZA is unaffected by ABT or the absence of NADPH, while MPZS is strongly inhibited by ABT and does not form in absence of NADPH. (B) (Lower) Peak Area Ratio of MPZ, MPZA, MPZS, and MPZH from same samples. MPZA is unaffected by CYP‐inhibition or lack of NADPH, while MPZH and MPZS are inhibited in the presence of ABT and are not produced in the absence of NADPH

**FIGURE 3:**
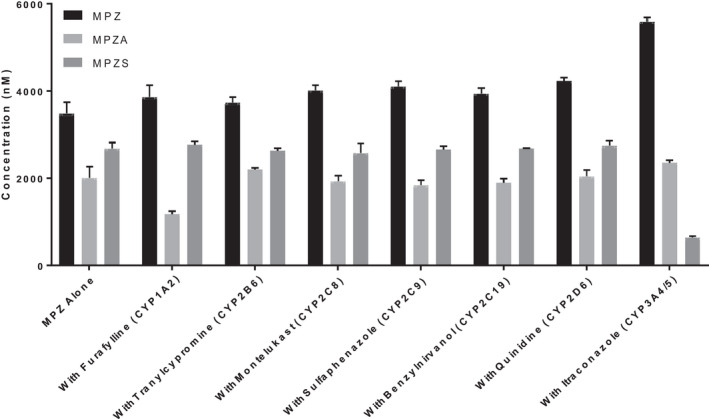
Mean (±SD) concentrations of MPZ, MPZA, and MPZS following 60‐min incubations of 1 µM MPZ with HLM each with specific inhibitors for seven common cytochrome P450 enzymes. MPZS formation was inhibited by the presence of the CYP3A4/5 inhibitor itraconazole

**FIGURE 4:**
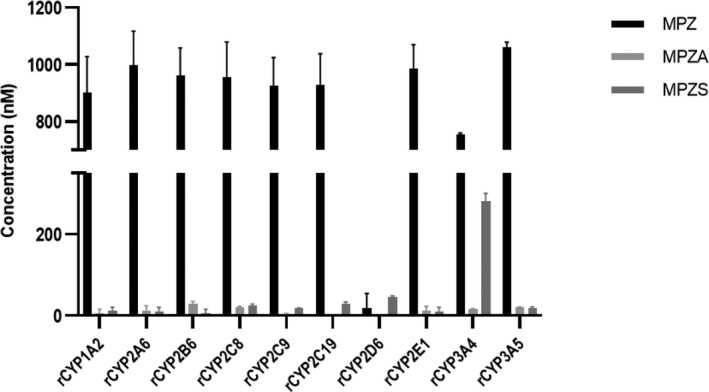
Mean (±SD) concentrations of MPZ, MPZA, and MPZS following 60‐min incubations of 1 µM MPZ with specific recombinant CYP isoforms. MPZS was produced mainly by CYP3A4 and not CYP3A5

To determine the involvement of cytochrome P450 enzymes, MPZ was incubated in HLM at 1 and 10 μM in the presence and absence of 1‐ABT, a known non‐specific CYP inhibitor, and with and without NADPH cofactor addition to the incubation mixture. As shown Figure 2A, the presence of 1‐ABT in the incubation inhibited the formation of MPZS but had no effect on MPZA. In addition, the absence of NADPH also did not reduce the formation of MPZA, but completely shut down the formation of MPZS. Looking qualitatively at peak area ration (PAR) in Figure 2B, MPZH formation, like MPZS, was also inhibited by the pan‐CYP inhibitor 1‐ABT and formation of MPZH was also completely shut down in the absence of NADPH. This indicates that NADPH‐ dependent cytochrome P450 enzymes are involved in the production of both the MPZS and MPZH metabolites; however, MPZA is not formed by cytochrome P450 enzymes, or any NADPH‐dependent metabolic process. To determine which CYP isoforms were involved in MPZ metabolism, separate HLM incubations were performed with specific inhibitors for seven common cytochrome P450 enzymes and the amount of MPZ, MPZA and MPZS quantified by mass spectrometry analysis. MPZS formation was clearly inhibited by the presence of the CYP3A4‐specific inhibitor itraconazole (Figure 3). None of these inhibitors affected MPZA formation. In addition, recombinant CYPs were incubated with MPZ for 60 min to confirm production of any metabolites. As shown in Figure 4, MPZS is mainly produced by CYP3A4. Looking qualitatively at PAR, MPZH formation was inhibited by the 2D6‐inhibitor quinidine and was only produced with recombinant CYP2D6 (data not shown). None of the other CYP enzymes appeared to have significant contribution to MPZ metabolism.

### 3.5 Enzymes involved in formation of MPZA

Since MPZA formation was not mediated by cytochrome P450 enzymes, other enzymes, FMO and hydroxylase, were investigated. To determine the contribution of microsomal FMOs on the metabolism of MPZ, incubations of MPZ were done in mixed, pooled HLM or HKM at 1 and 10 µM in triplicate in the presence of NADPH at pH 8.4 (a pH that should minimize CYP involvement) with and without an FMO inhibitor (methimazole). An HLM control was included in which HLM was pre‐incubated at 50°C for 5 min to inactivate CYP and FMO activities. Considerable formation of MPZA was observed with all conditions in HLM at pH 8.4 and was increased with increasing concentration of MPZ.

**FIGURE 5:**
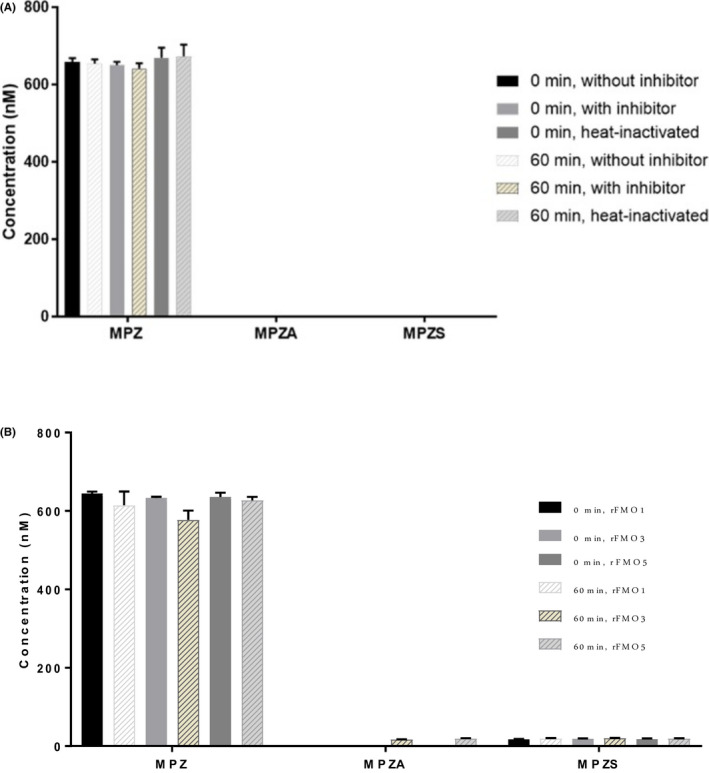
(A) (Upper) Mean (±SD) concentrations of metopimazine, MPZA and MPZS following incubations of 1 µM metopimazine in mixed, pooled HKM in triplicate in the presence of NADPH at pH 8.4. Heat‐inactivation (50°C for 5 min) and the presence and absence of the FMO inhibitor, methimazole, were all tested. No metabolites were produced under any condition. (B) (Lower) Mean (±SD) concentrations of MPZ, MPZA, and MPZS following incubations at 1 µM MPZ with recombinant FMO enzymes

Incubation of MPZ with HKM at pH 8.4 did not produce any metabolites (Figure 5A). Together, these results indicate that liver metabolism is unaffected by pH, FMO‐ inhibition, or even high temperature incubation, while there is no evidence of metabolism by HKM. The lack of formation of any metabolites at pH 8.4 in the HKM incubations demonstrates that the formation of MPZA is not catalyzed by pH alone. Furthermore, MPZA formation in HLM was not inhibited by a known FMO inhibitor, methimazole or heat (FMO inactivated), ruling out the involvement of FMO enzymes in the MPZA metabolite formation. Consistent with this, recombinant flavin monooxygenases enzymes (rhFMO1, rhFMO3, and rhFMO5) did not metabolize MPZ upon incubation (Figure 5B).

**FIGURE 6:**
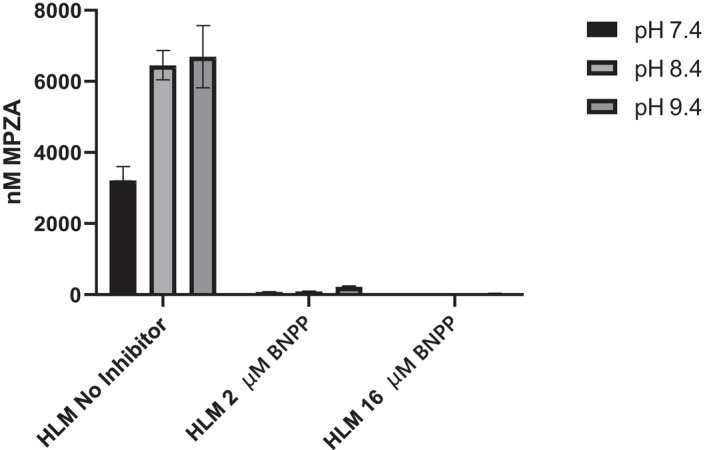
Formation of MPZA in nM (Mean ± SD) following 10 µM metopimazine incubation in HLM over 60 min in the absence and presence of 2 µM and 16 µM BNPP, a known amidase inhibitor, at pH 7.5, 8.4, and 9.4. Production of MPZA occurs at all pHs and increases at pH’s above physiologic pH, including pH 9.4. The amidase inhibitor BNPP effectively inhibits formation of MPZA under all conditions

In order to track down what hydrolase activity was responsible for the metabolism of MPZ to MPZA, an HLM incubation was performed under previous conditions except at increasing pH values 7.4, 8.4 and 9.4. MPZA formation was actually faster the higher the pH, including very strong formation at pH 9.4, consistent with amidase activity (Figure 6). In addition, bis‐p‐nitrophenyl phosphate (BNPP), an amidase inhibitor, was also added to some of the incubations. When this amidase inhibitor was present, at least 97% of all activity was inhibited at 2 μM, even at the highest and most active pH conditions. When this inhibitor concentration was increased to 16 μM, >99% of MPZA formation was inhibited at all three pH values. Taken together, these results are consistent with MPZA being formed by a human microsomal liver amidase.

The formation of MPZA was also observed in the incubation of 10 μM MPZ in human liver cytosol (HLC). HLC catalyzed the formation of some MPZA over 60 min of incubation, which was inhibited (48%) when 100 μM menadione, a known inhibitor of human AO, was included in the incubation (Table 5). These findings suggests that human liver cytosolic aldehyde oxidase is a potential contributor to the MPZA pathway.

**TABLE 5:** Concentrations of metopimazine acid in pooled human cytosolic incubations without and with menadione (AO Inhibitor)

Incubation time (min)	Metopimazine acid formation (nM)		
	No menadione	100 µM menadione	% Inhibited
0	0.00	0.00	47.59%
60	1371.66 ± 70.93	718.90 ± 21.40	

Abbreviation: BQL, below quantifiable limit.

These results clearly demonstrate a plausible MPZ metabolic scheme in which the major in vivo metabolite, MPZA, is catalyzed by a liver amidase with some potential contribution from aldehyde oxidase. The oxidative metabolites are catalyzed by CYP3A4 and CYP2D6 in vitro but are very minor pathways in vivo and have a negligible impact on human metabolism and clearance (Figure 5).

## 4 DISCUSSION

Clinical pharmacokinetic studies with MPZ have demonstrated that MPZA is the major in vivo metabolite in humans and based on AUC exposure, it accounts for over 90% of circulating drug‐related material.^9^ MPZA is a disproportionate metabolite, produced in far lower percentages in nonclinical species. Despite the length of time that MPZ has been an approved drug in France, little was known about the enzymes responsible for MPZ metabolism. A logical first step in elucidating the metabolic pathway of MPZ was to start with the cytochrome P450 (CYP) enzymes class. This superfamily of hemoproteins accounts for the majority of drug metabolism reactions.^10^ Regulatory agencies require the investigation of the involvement of major CYP isoforms including CYP3A4 and CYP2D6.^11^ Our studies have shown that CYP involvement is minimal in MPZ metabolism, with MPZS produced mainly by CYP3A4 and MPZH was only produced by CYP2D6, in vitro. MPZS was verified to be present in human plasma samples at very low concentrations and represented a small fraction of circulating drug‐related material (< 1%) based on AUC. MPZH does not appear to be a significant human metabolite. Importantly, no CYPs are involved with MPZA formation.

In this study, MPZ was shown to undergo hydrolysis in human liver microsomes to form MPZA. This conversion of MPZ to MPZA is not inhibited by any individual CYP inhibitor, including ABT, a non‐specific inhibitor of CYPs. This activity is not dependent on NADPH and increases under conditions above physiologic pH. Amidase/hydrolases, such as fatty acid amide hydrolase (FAAH) are known to have optimal activity in the pH range of 8.5–10.^12^, ^13^, ^14^ In HLM, the microsomal conversion of MPZ to MPZA was maximal at the highest pH tested, pH 9.4. These characteristics clearly point to a liver amidase as the enzyme responsible for MPZA formation. Consistent with these findings, the addition of BNPP, a known amidase inhibitor,^15^, ^16^ dramatically inhibited the formation of MPZA in HLM.

There are over 100 human genes that encode hydrolases that include esterases, amidases, peptidases and other enzymes.^17^ For prodrugs, hydrolytic activation by non‐CYP enzymes is the norm.^18^, ^19^ In an examination of 22 pro‐drugs approved by the FDA between 2006 and 2015, 19 underwent metabolic bioactivation via a “hydrolase,” which encompassed the broad category of esterases, amidases and other hydrolytic activities.^18^ When prodrugs are excluded, however, non‐CYP metabolism is not as common as biotransformation by CYP enzymes. Looking at this same time period but excluding prodrugs, 30% of the 125 FDA‐approved drugs were metabolized by non‐P450 enzymes compared to over half that were.^18^ Not surprisingly, conjugation by uridine 5’‐diphospho‐glucuronosyltransferases (UGTs) was the top non‐CYP activity but it was followed surprisingly closely by hydrolase activity (11.7% vs. 10.8%, for UGTs and hydrolases, respectively). Of the non‐prodrugs that were metabolically hydrolyzed, amidase activity accounted for 84%.^18^

Amidases, a class of hydrolase enzymes, are ubiquitous and their functions vary widely.^20^, ^21^ They hydrolyze a wide variety of amides including short‐ and mid‐chain aliphatic amides, arylamides, α‐aminoamides, and α‐ hydroxyamides. The catalytic mechanism of amidases involves the formation and hydrolysis of a covalent intermediate and adds water to the substrate without requiring additional cofactors. Amidases characteristically resist denaturation at higher pHs and temperatures, in stark contrast to CYP enzymes, likely due to the compact multimeric structure of amidases. Amidases such as Fatty Acid Amide Hydrolase (FAAH) are known to hydrolyze endogenous substrates such as endocannabinoid anandamide and oleamide.^22^ In vitro, FAAH can also cleave a variety of lipid amides^23^ and inhibition of FAAH‐1 has been used as a therapeutic strategy.^24^ However, amidases are not well defined in drug metabolism, despite the many amide‐containing drugs that undergo hydrolysis.^21^

While liver amidase conversion appears to be the primary biotransformation enzyme involved in MPZ metabolism, another enzyme that may contribute to the formation of MPZA is the cytosolic enzyme AO. We observed the conversion of MPZ to MPZA in HLC, though to a much lesser extent than HLM. This activity was moderately inhibited by menadione, a known inhibitor of AO, suggesting the possible contribution of AO to MPZ metabolism. AO is a metalloenzyme that contains molybdenum cofactor and typically shows hydroxylase activity.^25^, ^26^ It is most extensively expressed in the liver but also in the GI tract as well as the kidney, lungs, and skin.^27^, ^28^ AO catalyzes the oxidation of a wide range of drugs and xenobiotics that contain aldehydes (to their corresponding carboxylic acids) or azaheterocycles to lactams.^29^ AO is involved in the metabolism of several clinically significant drugs such as famciclovir, zaleplon, zonisamide, and ziprasidone.^17^ Association of AO with pathophysiology of a number of clinical disorders such as amyotrophic lateral sclerosis and alcohol induced liver injury, has been suggested.^30^ There is at least one published case of AO having amide hydrolase activity. Amide hydrolysis is the primary human metabolic clearance pathway for GDC‐0834, a Bruton's tyrosine kinase inhibitor; however, it is produced in much lower quantities in preclinical species.^31^ In humans, this amide hydrolase is catalyzed by AO.^32^

Although the hydrolysis of the amide bond in metopimazine appears to be catalyzed to a greater degree by a microsomal liver amidase, cytosolic AO can catalyze this reaction in vitro. While AO is known to catalyze a wide variety of biotransformations that include both oxidase activity and reductase activity,^30^ this is the second example in recent years of an atypical AO‐catalyzed amide hydrolysis of a small molecule and may indicate an expanding role for AO in xenobiotic metabolism.

We have demonstrated that the formation of MPZA from MPZ is primarily catalyzed by human liver amidase, with the potential for contribution from human liver cytosolic aldehyde oxidase, however to a much lesser extent. Clinical studies have demonstrated that conversion of MPZ to MPZA is by far the major metabolic process in humans, although this is not the case in the rat and the dog. Human liver cytochrome P450 enzymes are not involved in the formation of this major metabolite; however, they do appear to convert MPZ to 2 minor metabolites in vitro. Analysis of clinical samples from subjects receiving a 60 mg single dose of metopimazine indicates that MPZS is a very minor circulating metabolite and if MPZH is present it is only in transient trace amounts.

**FIGURE 7:**
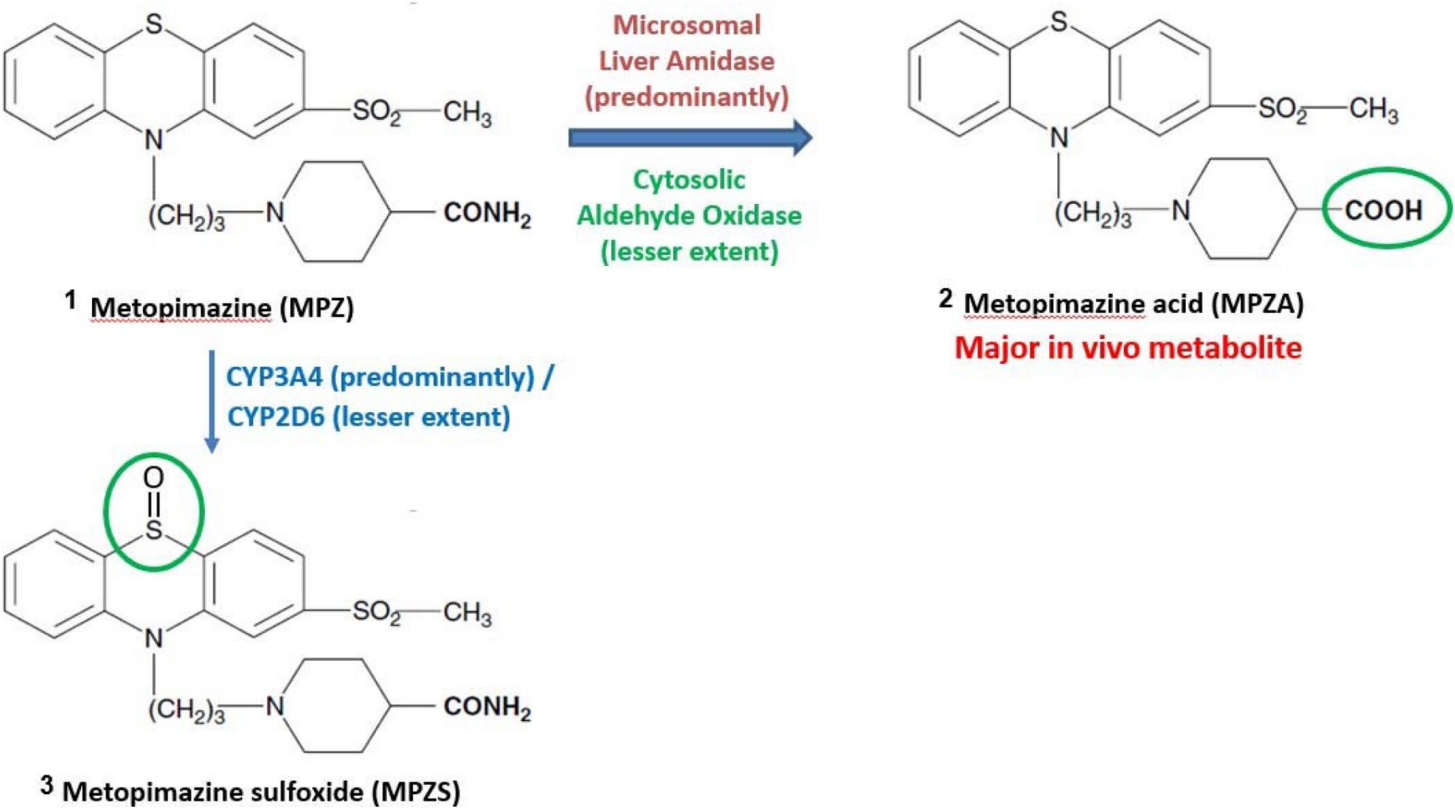
Metabolism of metopimazine in humans showing the formation of the major in vivo metabolite, MPZA, formed primarily by a liver amidase and to a lesser extent by cytosolic aldehyde oxidase. Trace amounts of the CYP‐mediated metabolite MPZS were also present in human samples

MPZS is predominantly catalyzed by CYP3A4. The metabolic scheme in humans is shown in Figure 7.

Liver amidases are not commonly thought of as drug metabolizing enzymes for small molecules and there is a dearth of literature on predicting human clearance when amide hydrolysis is the principal metabolic route. As additional examples of amide hydrolysis appear, species differences may be better understood and potential in vitro to in vivo extrapolation (IVIVE) approaches may be developed. In the case of metopimazine, the fact that amide hydrolysis is the main step in human MPZ metabolism contrasts markedly with other drugs used or in development for the treatment of GP. This may make combination and concomitant therapies more feasible for the treatment of GP with less drug‐drug interaction possibilities.

## DISCLOSURE

Dr. Busby has received consulting fees from Neurogastrx. Drs. De Colle and Wax have stock options in Neurogastrx.

## AUTHOR’S CONTRIBUTIONS

Busby, De Colle, Sadeque, Shen participated in research design. Cai, Yang, and Ramos conducted experiments. Cai, Venkatarangan, and Sadeque performed data analysis. Busby, Cai, De Colle, Sadeque, Shen, Wax and Yang wrote or contributed to the writing of the manuscript.

## ETHICS STATEMENT

This paper is the original and authentic work of the authors. All authors read and approved the final manuscript.

## Data Availability

The data that support the findings of this study are available from the corresponding author upon reasonable request.

## References

[1] Camilleri M , Parkman HP , Shafi MA , Abell TL , Gerson L . Clinical guideline: management of gastroparesis. Am J Gastroenterol. 2013;108:18‐38. 23147521 10.1038/ajg.2012.373 PMC3722580

[2] De Colle C , Van der Hart M , Chen J , Rassoulpour A , Pasricha PJ . 1079 NG101: a potent and selective dopamine D2 receptor antagonist as a potential alternative to metoclopramide and domperidone for the treatment of gastroparesis. Gastroenterology. 2016;150(4):S214.

[3] Stein B , Everhart KK , Lacy BE . Gastroparesis a review of current diagnosis and treatment options. J Clin Gastroenterol. 2015;49:550‐558. 25874755 10.1097/MCG.0000000000000320

[4] Herrstedt J , Jørgensen M , Angelo HR . The effect of food on serum concentrations of metopimazine. Br J Clin Pharmacol. 1990;30:237‐243. 2206785 10.1111/j.1365-2125.1990.tb03770.x PMC1368223

[5] Mallet E , Bounoure F , Skiba M , Saussereau E , Goulle J‐P , Castranet M . Pharmacokinetic study of metopimazine by oral route in children. Pharmacol Res Perpect. 2015;3(3):1‐7. 10.1002/prp2.130 PMC4492748 26171218

[6] Schoenberger JA . Drug‐induced orthostatic hypotension. Drug Saf. 1991;6:402‐407. doi:10.2165/00002018-199106060-00002 1793521

[7] Harding SD , Sharman JL , Faccenda E , et al. The IUPHAR/BPS Guide to PHARMACOLOGY in 2019: updates and expansion to encompass the new guide to IMMUNOPHARMACOLOGY. Nucleic Acids Res. 2018;46:D1091‐1106. doi:10.1093/nar/gkx1121 29149325 PMC5753190

[8] Alexander SPH , Christopoulos A , Davenport AP , et al. The Concise Guide to PHARMACOLOGY 2019/20: G protein‐coupled receptors. Br J Pharmacol. 2019;176(S1):S21‐S141. 31710717 10.1111/bph.14748 PMC6844580

[9] Herrstedt J , Jørgensen M , Angelo HR . Bioavailability of the antiemetic metopimazine given as a microenema. Br J Clin Pharmacol. 1996;41:613‐615. 8799530 10.1046/j.1365-2125.1996.35220.x PMC2042615

[10] Lynch T , Price A . The effect of cytochrome P450 metabolism on drug response, interactions, and adverse effects. Am Fam Physician. 2007;76:391‐396. 17708140

[11] US FDA . Guidance for industry – in vitro drug interaction studies – cytochrome p450 enzyme‐ and transporter‐mediated drug interactions. 2020.

[12] Asano Y , Achibana MT , Ani YT , Yamada H . Purification and characterization of amidase which participates in nitrile degradation. Agric Biol Chem. 1982;46(5):1175‐1181.

[13] Bisogno T , Maurelli S , Melck D , De Petrocellis L , Di Marzo V . Biosynthesis, uptake, and degradation of anandamide and palmitoyl ethanolamide in leukocytes. J Biol Chem. 1997;272:3315‐3323. 9013571 10.1074/jbc.272.6.3315

[14] Ueda N , Yamanaka K , Yamamoto S . Purification and characterization of an acid amidase selective for *N*‐palmitoylethanolamine, a putative endogenous anti‐ inflammatory substance. J Biol Chem. 2001;276:35552‐35557. 11463796 10.1074/jbc.M106261200

[15] Sarich TC , Adams SP , Petricca G , Wright JM . Inhibition of isoniazid‐induced hepatotoxicity in rabbits by pretreatment with an amidase inhibitor. J Pharmacol Exp Ther. 1999;289:695‐702. 10215642

[16] Shih T , Pai C , Yang P , Chang W , Wang N , Hu OY . A novel mechanism underlies the hepatotoxicity of pyrazinamide. Antimicrob Agents Chemother. 2013;57:1685‐1690. 23357778 10.1128/AAC.01866-12 PMC3623344

[17] Parkinson A , Ogilvie BW , Buckley DB , Kazmi F , Parkinson O . Chapter 6: biotransformation of xenobiotics. In: Klaassen C , ed. Casarett and Doull's Toxicology: The Basic Science of Poisons. 9th ed. McGraw‐Hill Education; 2019:193‐399.

[18] Cerny MA . Prevalence of non‐cytochrome P450‐mediated metabolism in food and drug administration‐approved oral and intravenous drugs: 2006–2015. Drug Metab Dispos. 2016;44:1246‐1252. 27084892 10.1124/dmd.116.070763

[19] Saravanakumar A , Sadighi A , Ryu R , Akhlaghi F . Physicochemical properties, biotransformation, and transport pathways of established and newly approved medications: a systematic review of the top 200 most prescribed drugs vs. the FDA‐ approved drugs between 2005 and 2016. Clin Pharmacokinet. 2019;58:1281‐1294. doi:10.1007/s40262-019-00750-8 30972694 PMC6773482

[20] Sharma M , Sharma NN , Bhalla TC . Amidases: versatile enzymes in nature. Rev Environ Sci Biotechnol. 2009;8:343. doi:10.1007/s11157-009-9175-x

[21] Testa B , Mayer JM . The hydrolysis of amides. In: Hydrolysis in Drug and Prodrug Metabolism. Verlag Helvetica Chimica Acta; 2006:81‐162.

[22] Cravatt BF , Giang DK , Mayfield SP , Boger DL , Lerner RA , Gilula NB . Molecular characterization of an enzyme that degrades neuromodulatory fatty‐acid amides. Nature. 1996;384:83‐87. 8900284 10.1038/384083a0

[23] Long JZ , Cravatt BF . The metabolic serine hydrolases and their functions in mammalian physiology and disease. Chem Rev. 2011;111:6022‐6063. 21696217 10.1021/cr200075y PMC3192302

[24] Alibanijamali AR , Wakefield JD , Mermerian AH , Busby RW . Metabolism and disposition of MM‐433593, a selective FAAH‐1 inhibitor, in monkeys. Pharmacol Res Perpect. 2014;2(5):e00059. doi:10.1002/prp2.59 PMC4186420 25505606

[25] Garattini E , Terao M . The role of aldehyde oxidase in drug metabolism. Expert Opin Drug Metab Toxicol. 2012;8:487‐503. 22335465 10.1517/17425255.2012.663352

[26] Mendel RR . Cell biology of molybdenum. BioFactors. 2009;35:429‐434. 19623604 10.1002/biof.55

[27] Abbasi A , Paragas EM , Joswig‐Jones CA , Rodgers JT , Jones JP . Time course of aldehyde oxidase and why it is nonlinear. Drug Metab Dispos. 2019;47:473‐483. 30787100 10.1124/dmd.118.085787 PMC6439458

[28] Garattini E , Fratelli M , Terao M . Mammalian aldehyde oxidases: genetics, evolution and biochemistry. Cell Mol Life Sci. 2008;65:1019‐1048. 18066686 10.1007/s00018-007-7398-y PMC11131919

[29] Beedham C . Molybdenum hydroxylases. In: Ioannides C , ed. Enzyme Systems that Metabolise Drugs and Other Xenobiotics. John Wiley & Sons; 2002:147‐187.

[30] Kitamura S , Sugihara K , Ohta S . Drug metabolizing ability of molybdenum hydrolases. Drug Metab Pharmacokinet. 2006;21(2):83‐98. 16702728 10.2133/dmpk.21.83

[31] Liu L , Halladay JS , Shin Y , et al. Significant species difference in amide hydrolysis of GDC‐0834, a novel potent and selective Bruton’s tyrosine kinase inhibitor. Drug Metab Dispos. 2011;39:1840‐1849. 21742900 10.1124/dmd.111.040840

[32] Sodhi JK , Wong S , Kirkpatrick DS , et al. A novel reaction mediated by human aldehyde oxidase: amide hydrolysis of GDC‐0834. Drug Metab Dispos. 2015;43:908‐915. 25845827 10.1124/dmd.114.061804 PMC4429680

